# Supplementary material for: A Triple Gene-Deleted Pseudorabies Virus-Vectored Subunit PCV2b and CSFV Vaccine Protects Pigs against PCV2b Challenge and Induces Serum Neutralizing Antibody Response against CSFV
Source: Vaccines (Basel). 2022 Feb 16;10(2):305. doi: 10.3390/vaccines10020305 (PMC8878206; doi:10.3390/vaccines10020305)
Supplement: Supplementary file 1 [file vaccines-10-00305-s001.zip › vaccines-1547187-supplementary.pdf]

5'-EcoRI

GAATTCtccgcagtaccggcgtcgatgatgatggtggcgcgcgacgtgacccggctccccgcggggctcc

PRV gE flanking upstream

tcctcgccgccctgaccctggccgccctgaccccgcgcgtcgggggcgtcctcttcaggggcgccggcgt
cagcgtgcacgtcgccggcagcgccgtcctcgtgcccggcgacgcgcccaacctgacgatcgacgggacg
ctgctgtttctggaggggccctcgccgagcaactacagcgggcgcgtggagctgctgcgcctcgacccca
agcgcgcctgctacacgcgcgagtacgccgccgagtacgacctctgcccccgcgtgcaccacgaggcctt
ccgcggctgtctgcgcaagcgcgagccgctcgcccggcgcgcgtccgccgcggtggaggcgcgccggctg
ctgttcgtctcgcgcccggccccgccggacgcggggtcgtacgtgctgcgggtccgcgtgaacgggacca
cggacctctttgtgctgacggccctggtgccgcccaggggggcgcccccaccaccccacgccgtcgtccgc
ggacgagtgccggcccgtcgtcggatcgtggcacgacagcctgcgcgtcgtggaccccgccgaggacgcc
gtgttcaccacgccgcccccgatcgagccagagccgccgacgacccccgcgcccccccgggggaccggcg
ccacccccgagccccgctccgacgaagaggaggaggacgaggaggggggcgacgacggcgatgaccccggt
gcccgggaccctggacgcgaacggcacgatggtgctgaacgccagcgtcgtgtcgcgcgtcctgctcgcc
gccgccaacgccacggcgggcgcccggggccccgggaagatagccatggtgctggggcccacgatcgtcg
tcctcctgatcttcttgggcggggtcgcctgcgcggcccggcgctgcgcgcggaatcgcatctaccggcc
gcgacccgggcgcggcccggcggtccacgcgccgcccccgcggcgcccgccccccagccccgtcgccggg
gcgcccgtcccccagcccaagatgacgttggccgagcttcgccagaagctggccaccatcgcagaggaac
aataaaaaggtggtgtttgcataattttgtgggtggcgttttatctccgtccg**GGTACC**ATAGTAATCAA

KpnI **CAG promotor**

TTACGGGGTCATTAGTTCATAGCCCATATATGGAGTTCCGCGTTACATAACTTACGGTAAATGGCCCGCC
TGGCTGACCGCCCAACGACCCCCGCCCATTGACGTCAATAATGACGTATGTTCCCATAGTAACGCCAATA
GGGACTTTCCATTGACGTCAATGGGTGGACTATTTACGGTAAACTGCCCACTTGGCAGTACATCAAGTGT
ATCATATGCCAAGTACGCCCCCTATTGACGTCAATGACGGTAAATGGCCCGCCTGGCATTATGCCCAGTA
CATGACCTTATGGGACTTTCCTACTTGGCAGTACATCTACGTATTAGTCATCGCTATTACCATGCGTCGA
GGTGAGCCCCACGTTCTGCTTCACTCTCCCCATCTCCCCCCCCTCCCCACCCCCAATTTTGTATTTATTT
ATTTTTTAATTATTTTATGCAGCGATGGGGGCGGGGGGGGGGGGGGCGCGCGCCAGGCGGGGCGGGGCGG
GGCGAGGGGCGGGGCGGGGCGAGGCGGAGAGGTGCGGCGGCAGCCAATCAGAGCGGCGCGCTCCGAAAGT
TTCCTTTTATGGCGAGGCGGCGGCGGCGGCGGCCCTATAAAAAGCGAAGCGCGCGGCGGGCGGGAGTCGC
TGCGTTGCCTTCGCCCCGTGCCCCGCTCCGCGCCGCCTCGCGCCGCCCGCCCCGGCTCTGACTGACCGCG
TTACTCCCACAGGTGAGCGGGCGGGACGGCCCTTCTCCTCCGGGCTGTAATTAGCGCTTGGTTTAATGA
CGGCTCGTTTCTTTTCTGTGGCTGCGTGAAAGCCTTAAAGGGCTCCGGGAGGGCCCTTTGTGCGGGGGGA
GCGGCTCGGGGGGTGCGTGCGTGTGTGTGTGCGTGGGGAGCGCCGCGTGCGGCCCGCGCTGCCCGGCGGC
TGTGAGCGCTGCGGGCGCGGCGCGGGGCTTTGTGCGCTCCGCGTGTGCGCGAGGGGAGCGCGGCCGGGG
GCGGTGCCCCGCGGTGCGGGGGGGCTGCGAGGGGAACAAAGGCTGCGTGCGGGGTGTGTGCGTGGGGGGG
TGAGCAGGGGGTGTGGGCGCGGCGGTCGGGCTGTAACCCCCCCCTGGCACCCCCCTCCCCGAGTTGCTGA
GCACGGCCCGGCTTCGGGTGCGGGGCTCCGTGCGGGGCGTGGCGCGGGGCTCGCCGTGCCGGGCGGGGGG
TGGCGGCAGGTGGGGGTGCCGGGCGGGGCGGGGCCGCCTCGGGCCGGGGAGGGCTCGGGGGAGGGGCGCG
GCGGCCCCGGAGCGCCGGCGGCTGTCGAGGCGCGGCGAGCCGCAGCCATTGCCTTTTATGGTAATCGTGC
GAGAGGGCGCAGGGACTTCCTTTGTCCCAAATCTGGCGGAGCCGAAATCTGGGAGGCGCCGCCGCACCCC
CTCTAGCGGGCGCGGGCGAAGCGGTGCGGCGCCGGCAGGAAGGAAATGGGCGGGGAGGGCCTTCGTGCGT
CGCCGCGCCGCCGTCCCCTTCTCCATCTCCAGCCTCGGGGCTGCCGCAGGGGGACGGCTGCCTTCGGGGG
GGACGGGGCAGGGCGGGGTTCGGCTTCTGGCGTGTGACCGGCGGCTTTAGAGCCTCTGCTAACCATGTTC
ATGCCTTCTTCTTTTTCCTACAGCTCCTGGGCAACGTGCTGGTTGTTGTGCTGTCTCATCATTTTGGCAA
gctagc*GCCGCCACC*ATGCTCCTCGCCGCTCTCCTCGCCGCTCTCGTCGCCAGAACCACACTCGGGGCCA

NheI **Kozak** **PRV gD signal sequence**

GACTCGCCTGTAAAGAAAATTATAGATACGCCATCTCCAGCACCAACGAGATTGGGCTGCTCGGCGCCGA

CSFV E2

AGGACTGACCACAACCTGGCAGGAGTACAACCAGGACCTGCAGCTCAATGATGGCACCGTCAAGGCCATC
TGCGAAGCTGGCTCCTTCAAAGTGATTGCTCTGAATGTGGTCTCTCGCCGCTACCTGGCCTCACTCCACA
AGGAGGCTTCTCTGACCTCAGTGACATTCGAACTGCTCTTTGACGGGACCTCCCCCAGCACAGAGGAAAT
GGGCGACGATTTCGGCTTTGGACTGTGTCCCTTCGATACCTCCCCAGTGGTCAAGGGAAAATACAACACA

ACCCTGCTCAATGGGAGCGCCTTTTATCTGGTGTGCCCAATCGGATGGACCGGAGTCATTGAGTGTACAG
CTGTGAGCCCAACAACCCTGAGGACCGAAGTGGTCAAGACATTCAGGAGAGACAAACCCTTTCCACACCG
CATGGATTGCATGACAACCACAGTGGAGAACGAAGACCTGTTCTACTGCAAGCTCGGCGGAAATTGGACC
TGTGTGAAAGGCGAGCCTGTGGTCTATACAGGGGGCCCCGTGAAGCAGTGCAAATGGTGTGGCTTCGACT
TTAACGAGCCTGATGGACTGCCACATTACCCTATCGGGAAGTGCATTCTCGCCAACGAGACCGGCTATAG
AATCGTCGACAGCACAGATTGCAATCGAGACGGCGTGATCATTTCCACCGAGGGAAGCCACGAATGTCTG
ATCGGGAACACCACAGTCAAAGTGCATGCCTCCGACGAGCGACTCGGACCTATGCCATGCCGACCAAAGG
AAATCGTCTCTTCAGCCGGACCTGTGCGCAAAACCTCTTGTACATTCAACTACGCTAAGACCCTGCGGAA
TAAATACTATGAGCCAAGGGACTCCTACTTCCAGCAGTATATGCTGAAGGGCGAATACCAGTATTGGTTT
GACCTCGATGTGACCGACCGGCATGCCGATTACTTCGCTGAGTTTGTGGTCCTGGTGGTCGTGGCCCTGC
TCGGAGGGAGATACGTCCTCTGGCTCATTGTGACATACATCGTCCTGACCGAACAGCTCGCCGCCGGG**cc**
atgg*ggtaagcctatccctaaccctctcctcggtctcgattctacg*CGTACCGGT**CATCATCACCATCAC**
NcoI **V5 epitope** **6x His**
CAT***TGA***GTTTAAACCCGCTGATCAGCCTCGA*CTGTGCCTTCTAGTTGCCAGCCATCTGTTGTTTGCCCCT*
Stop **Poly A**
CCCCCGTGCCTTCCTTGACCCTGGAAGGTGCCACTCCCACTGTCCTTTCCTAATAAAATGAGGAAATTGC
ATCGCATTGTCTGAGTAGGTGTCATTCTATTCTGGGGGGTGGGGTGGGGCAGGACAGCAAGGGGGAGGAT
*TGGGAAGACAATAGCAGGCATGCTGGGGATGCGGTGGGCTCTATGG*CTT**GGATCC**atgcccgccgccgcc
BamHI
cccgccgacatggacacgttcgaccccagcgcccccgtcccgacgagcgtctctaacccggccgccgacg
PRV gE flanking downstream
tcctgctggcccccaagggaccccgctccccgctgcgcccccaggacgactcggactgctactacagcga
gagcgacaacgagacgcccagcgagttcctgcgccgcgtgggacgccggcaggcggcgcgccggagacgc
cgccgctgcctgatgggcgtcgcgatcagcgccgccgcgctggtcatctgctcgctgtcggcgctgatcg
ggggcatcatcgcccggcacgtgtagcgagcgggtggtggccgcccgccccgccgcgcccaggaggggg
gtccgggggggcgaagcgggcggaggagagcgagccacgtggttgtgggctcggacttgtcacaataaat
gggccccggcgcacccgggcgcacacagcagccttcctcgtctccgcgtctctgctgttcctctcgtcgg
tcttctcccactccgccgtcgcgaacgcgctcgcgccatgggggtgacggccatcaccgtggtcacgctg
atggacgggtccgggcgcatccccgccttcgtgggcgaggcgcacccggacctgtggaaggtgctcaccg
agtggtgctacgcgtcgctggtgcagcagcggcgcgccgccgacgaggacacgccgcggcaacacgtggt
gctgcgctcctcggagatcgcccccggctcgctggccctgctgccgcgcgccacgcgccccgtcgtgcgg
acacggtccgaccccacggcgccgttctacatcaccaccgagacgcacgagctgacgcggcgcccccccgg
cggacggctcgaagcccggggagcccctccgtatcagcccgcccccgcggctggacacggagtggtcctc
cgtcatcaac**AAGCTT**-3
HindIII

Supplementary figure S1: Nucleotide sequence of the PRV gEΔ CSFV E2 insertion plasmid (pPRV gEΔ/CSFV E2-INS) with pCAG promotor. The nucleotide sequence for the PRV gEΔ CSFV E2 insertion was assembled using the plasmid pPRV gEΔ and pCSFV E2 chimeric gene cassette. The PRV gEΔ CSFV E2 insertion plasmid consisted of the following (5’ to 3’): i) EcoRI restriction site (bold, underlined); ii) PRV gE flanking upstream (lower case); iii) KpnI restriction site (bold, underlined); iv) CAG promotor sequence; v) NheI restriction site (bold, underlined); vi) Kozak sequence (italic); vii) PRV gD signal (underlined); viii) CSFV E2 coding sequence (highlighted); ix) NcoI restriction site (bold, underlined); x) V5 epitope coding sequence (lower case, italic); xi) 6x His epitope coding sequence (bold); xii) Stop codon (TGA) (bold, italic); xiii) bovine growth hormone terminator (italic, underlined); xiv) BamHI restriction site (bold, underlined); xv) PRV gE flanking downstream (lower case); xvi) HindIII restriction site (bold, underlined).

5’-EcoRI

GAATTCccgccttatcaccgcgcccacccgctccccgcccggccccgcgcgcagcgatcgcga**tca**cc

PRV TK flanking upstream **Stop U_L24**

gccgcggcccggcgacgtactcggcgaggccgcgcacggtcgcggccatcgcgctcgcgttgccgcgcgt
ctgggtgcagggcaggcgcgtcacgtcgagcacgcgcatgctccgctgggccacgaacaccagcaggggc
acgagcgtgatctcctcgccgcccgggggcacggcggcggcgaggaggcgcgccgagtcgcgcagctggc
acagcccctcgtgccgctgcccgcgcttgctgggcgtgttgaggttccgggggaagcggcacgtcttgag
ctcgatgacgaagcacaggtgcggcccacccccagccgcaccacgcacacgcagtcggggcggcgcacc
ccgaggttgacttcaaaggccagggtcaaggacgccttcttaagcgtctcgcggggaagcccgaagagac
tctcgccgtacgcggacgggtcgcggcgcaggcgttcgtagaagcggttgtggcagcggatccccgcccg
gaagcgcgccggg**atg**cg**cat**cctcaggatctacctcgacggcgcctacggcaccggcaagagcaccacg

Start TK → **←Start U_L24**

Gcccgggtgatggcgctcggcggggcgctgtacgtgctcgagccgatggcgtactggcgcactctgttcg
acacggacacggtggccggtatttacgatgcgcagacccggaagcagaacggcagcctgagcgaggagga
cgcggccctcgtcacggcgcagcaccaggccgccttcgcgacgccgtacctgctgctgcacacgcgcctg
gtcccgctcttcgggcccgcggtcgagggcccgcccgtgatgacggtcgtctttgaccgccacccggtgg
ccgcgacggtgtgcttcccgctggcgcgcttcatcgtcggggacatcagcgcggcggccttcgtgggcTA
AGTGAGTAA**GGTACC**CTCGTGAGGCTCCGGTGCCCGTCAGTGGGCAGAGCGCACATCGCCCACAGTCCCC

Stop/Stop/Stop KpnI **hEF-1α promoter**

GAGAAGTTGGGGGGAGGGGTCGGCAATTGAACCGGTGCCTAGAGAAGGTGGCGCGGGGTAAACTGGGAAA
GTGATGTCGTGTACTGGCTCCGCCTTTTTCCCGAGGGTGGGGGAGAACCGTATATAAGTGCAGTAGTCGC
CGTGAACGTTCTTTTTCGCAACGGGTTTGCCGCCAGAACACAGGTAAGTGCCGTGTGTGGTTCCCGCGGG
CCTGGCCTCTTTACGGGTTATGGCCCTTGCGTGCCTTGAATTACTTCCACCTGGCTGCAGTACGTGATTC
TTGATCCCGAGCTTCGGGTTGGAAGTGGGTGGGAGAGTTCGAGGCCTTGCGCTTAAGGAGCCCCTTCGCC
TCGTGCTTGAGTTGAGGCCTGGCCTGGGCGCTGGGGCCGCCGCGTGCGAATCTGGTGGCACCTTCGCGCC
TGTCTCGCTGCTTTCGATAAGTCTCTAGCCATTTAAAATTTTTGATGACCTGCTGCGACGCTTTTTTTCT
GGCAAGATAGTCTTGTAAATGCGGGCCAAGATCTGCACACTGGTATTTCGGTTTTTGGGGCCGCGGGCGG
CGACGGGGCCCGTGCGTCCCAGCGCACATGTTCGGCGAGGCGGGGCCTGCGAGCGCGGCCACCGAGAATC
GGACGGGGGTAGTCTCAAGCTGGCCGGCCTGCTCTGGTGCCTGGCCTCGCGCCGCCGTGTATCGCCCCGC
CCTGGGCGGCAAGGCTGGCCCGGTCGGCACCAGTTGCGTGAGCGGAAAGATGGCCGCTTCCCGGCCCTGC
TGCAGGGAGCTCAAAATGGAGGACGCGGCGCTCGGGAGAGCGGGCGGGTGAGTCACCCACACAAAGGAAA
AGGGCCTTTCCGTCCTCAGCCGTCGCTTCATGTGACTCCACGGAGTACCGGGCGCCGTCCAGGCACCTCG
ATTAGTTCTCGAGCTTTTGGAGTACGTCGTCTTTAGGTTGGGGGGAGGGGTTTTATGCGATGGAGTTTCC
CCACACTGAGTGGGTGGAGACTGAAGTTAGGCCAGCTTGGCACTTGATGTAATTCTCCTTGGAATTTGCC
CTTTTTGAGTTTGGATCTTGGTTCATTCTCAAGCCTCAGACAGTGGTTCAAAGTTTTTTTCTTCCATTTC
AGGTGTCGTGAGGAATTAGCTTGGTACTAATACGACTCACTATAGGGAGACCCAAGCTGGCTAGGTAAGT
GTACGAGCTCGATCACTAGTCCAGTGTGG**gctagc***GCCGCCACC*ATGCTGCTCGCCGCCCTGCTCGCTGC

NheI **Kozak** **PRV gD signal sequence**

CCTCGTCGCCCGCACAACACTGATGACATACCCTCGGAGAAGATACAGAAGAAATGGATTTTCAACACC

PCV2 Cap

AGGCTCTCTAGAACCTTTGGGTATACAGTGAAGCGCACCACAGTCACCACACCTTCCTGGCCGTGGACA
TGATGCGGTTCAAGCTGGACGATTTTGTCCCCCCTGGCGGGGGAACCAATAAAATCAGCATTCCCTTCGA
GTACTATCGCATCCGGAAGGTGAAAGTCGAATTTTGGCCCTGCTCCCCTATTACCCAGGGAGACAGGGGA
GTGGGATCTACAGCTGTCATCCTCGACGATAACTTCGTGCCAAAAGCCAATGCTCTGACCTACGATCCCT
ATGTGAACTACAGCTCCAGGCACACCATTCCACAGCCCTTCTCTTATCATAGCAGATACTTTACCCCTAA
GCCAGTGCTCGACTCCACAATCGATTACTTCCAGCCCAACAATAAACGCAACCAGCTGTGGCTCAGGCTG
CAGACCAGCCGGAATGTGGACCACGTCGGACTGGGCACAGCCTTTGAGAACTCCAAGTATGACCAGGATT
ACAATATCCGCGTCACAATGTATGTCCAGTTCAGGGAGTTCAATCTCAAAGACCCACCTCTCAATCCA**gc**
ggccgct*ggtaagcctatccctaaccctctcctcggtctcgattctacg*CGTACCGGT**CATCATCACCAT**

NotI → **V5 epitope** **6x His**

CACCAT*TGA*GTTTAAACCCGCTGATCAGCCT*CGACTGTGCCTTCTAGTTGCCAGCCATCTGTTGTTTGCC*

Stop **Poly A**

CCTCCCCCGTGCCTTCCTTGACCCTGGAAGGTGCCACTCCCACTGTCCTTTCCTAATAAAATGAGGAAAT
TGCATCGCATTGTCTGAGTAGGTGTCATTCTATTCTGGGGGGTGGGGTGGGGCAGGACAGCAAGGGGGAG
*GATTGGGAAGACAATAGCAGGCATGCTGGGGATGCGGTGGGCTCTATGG*CTT**AAGCTT**tcgctggacccg
HindIII
gacgagcacctgcggcgcctgcgcgcccgcgcgcgcgccggggagcacgtggacgcgcgcctgctcacgg
PRV TK flanking downstream
ccctgcgcaacgtctacgccatgctggtcaacacgtcgcgctacctgagctcggggcgccgctggcgcga
cgactgggggcgcgcgccgcgcttcgaccagaccacgcgcgactgcctcgcgctcaacgagctctgccgc
ccgcgcgacgaccccgagctccaggacaccctcttcggcgcgtacaaggcgcccgagctctgcgaccggc
gcgggcgcccgctcgaggtgcacgcgtgggcgatggacgcgctcgtggccaagctgctgccgctgcgcgt
ctccaccgtcgacctggggccctcgccgcgcgcctgcgccgcggccgtggcggcgcaggcgcgcggcatg
gaggtgacggagtccgcgtacggcgaccacatccggcagtgcgtgtgcgccttcacgtcggagatggggg
tg**tga**ccctcgcccctcccacccgcgccgcggccggatggagaccgcgacggaggcaacgacgacggcgt
Stop TK
Gggagggggctcggggcgcgtataaagccatgtgtatgtcatcccaataaagtttgccgtgcccgtcacc
atgcccgcgtcgtccgtgcgcctcccgctgcgcctcctgaccctcgcgggcctcctggccctcgcgggggg
Start U_L22 (gH) →
ccgccgccctcgcccgcggcgcgccgcagggtggccgccctcgccgcagggggtcccgcgcccaccgc
ggcgcccgcgcgcgggcccaccctgttcgtcccggtcggcgacggctccgcgtggttcgtcttccagctc
ggcgggctgggggcgctcaacgacacgcgcatccgcgggcacctgctcggcc**ATGCAT**-3
NsiI

Supplementary figure S2: Nucleotide sequence of the PRV TKΔ PCV2 Cap insertion plasmid (pPRV TKΔ/PCV2 Cap-INS). The nucleotide sequence for the PRV TKΔ PCV2 Cap insertion was assembled using the plasmid pPRV TKΔ and pPCV2 Cap chimeric gene cassette. The PRV TKΔ PCV2 Cap insertion plasmid consisted of the following (5' to 3'): i) EcoRI restriction site (bold, underlined); ii) PRV TK flanking upstream (lower case), start and stop codon of U_L24 and start codon of TK are highlighted; iii) three stop codons (underlined); iv) KpnI restriction site (bold, underlined); v) hEF-1α promotor sequence; vi) NheI restriction site (bold, underlined); vii) Kozak sequence (italic); viii) PRV gD signal (underlined); ix) PCV2 Cap coding sequence (highlighted); x) NotI restriction site (bold, underlined); xi) V5 epitope coding sequence (lower case, italic); xii) 6x His epitope coding sequence (bold); xiii) Stop codon (TGA) (bold, italic); xiv) bovine growth hormone terminator (italic, underlined); xv) HindIII restriction site (bold, underlined); xvi) PRV TK flanking downstream (lower case), stop codon of TK and start codon of U_L22 (gH) are highlighted; xvii) NsiI restriction site (bold, underlined).

5’- **EcoRI**

GAATTCggcgacggcgacggcgacagcagcggggacgaggacgacgatgacggggggctgacgcggcagg

PRV gG flanking upstream

ccgcgtcgcgcatcgccacggacctgggcttcgaggtgctgcagcccctgcagtcgggctcggagggccg
cgtcttcgtggcccgccggcccggcgaggcggacacggtggtgctgaaggtgggccagaagccctcgacg
ctgatggagggcatgctgctgaagcgcctggcccacgataacgtcatgagcctgaagcagatgctcgccc
ggggcccggtgacgtgcctggtcctgccgcactttcggtgcgatctgtacagctacctgaccatgcggga
cgggccgctggacatgcgcgacgccgggcgcgtgatccggtccgtgctccgcgggctcgcctacctgcac
gggatgcgcatcatgcaccgcgacgtcaaggcggagaacatcttcctcgaggacgtggacacggtgtgcc
tgggggacctcggggccgcgcgctgcaacgtggcggcgcccaacttttacggactcgccgggaccatcga
gaccaacgcccccgaggtgctcgcgcgcgaccgctacgacaccaaggtcgacgtctggggcgcgggggtg
gtgctcttcgagacgctggcctaccccaagacgatcgccggcggggacgagcccgcgatcaacggggaga
tgcacctgatcgacctcatccgcgccctcggggtgcaccccgaggagttcccgcccgacacgcgcctccg
gagcgagttcgtccggtacgccgggacccaccgccagccgtacacgcagtacgcgcgcgtggctcgcctc
gggctgcccgagacgggggctttcctgatttacaagatgttgacgtttgatcccgtccgccgcccttccg
ctgatgagatactcaactttggaatgtggaccgtataaaacggcccggctccgagcggtaggacacacac
acctttgcgcatctccacagctcaaca***ttcaataaagta***<u>**GGTACC**</u>TAGTTATTAATAGTAATCAATTACG

Us3/Us4 Poly A **KpnI** **CMV promotor**

GGGTCATTAGTTCATAGCCCATATATGGAGTTCCGCGTTACATAACTTACGGTAAATGGCCCGCCTGGCT
GACCGCCCAACGACCCCCGCCCATTGACGTCAATAATGACGTATGTTCCCATAGTAACGCCAATAGGGAC
TTTCCATTGACGTCAATGGGTGGAGTATTTACGGTAAACTGCCCACTTGGCAGTACATCAAGTGTATCAT
ATGCCAAGTACGCCCCCTATTGACGTCAATGACGGTAAATGGCCCGCCTGGCATTATGCCCAGTACATGA
CCTTATGGGACTTTCCTACTTGGCAGTACATCTACGTATTAGTCATCGCTATTACCATGGTGATGCGGTT
TTGGCAGTACATCAATGGGCGTGGATAGCGGTTTGACTCACGGGGATTTCCAAGTCTCCACCCCATTGAC
GTCAATGGGAGTTTGTTTTGGCACCAAAATCAACGGGACTTTCCAAAATGTCGTAACAACTCCGCCCCAT
TGACGCAAATGGGCGGTAGGCGTGTACGGTGGGAGGTCTATATAAGCAGAGCTGGTTTAGTGAACCGTCA
GATCCGCTAGCGCTACCGGT***GCCGCCACC***<u>ATGCTGCTCGCTGCTCTCCTCGCTGCTCTCGTCGCTCGCAC</u>

Kozak **PRV gD signal**

<u>AACCCTCGGGGCT</u>GAAAACATTACCCAGTGGAACCTCTCTGACAACGGGACCAACGGAATCCAGCACGCC

CSFV E^{rns} Brescia

ATGTACCTGAGGGGGGTGAGCAGATCCCTCCATGGAATCTGGCCAGAGAAGATTTGCAAAGGGGTCCCCA
CCTATCTGGCCACCGACACAGAGCTCAAGGAAATTCAGGGCATGATGGATGCTTCCGAGGGGACCAACTA
CACATGCTGTAAGCTGCAGCGGCACGAATGGAACAAGCATGGCTGGTGTAACTGGTATAATATCGACCCC
TGGATTCAGCTGATGAACAGGACCCAGGCTAATCTCGCTGAGGGACCACCTGCTAAGGAATGCGCTGTGA
CCTGTCGGTACGACAAAAACGCCGATATCAATGTGGTCACCCAGGCTAGGAACAGGCCAACCACACTGAC
AGGATGCAAGAAAGGCAAGAATTTCAGCTTTGCCGGGACCGTGATCGAGGGACCTTGCAACTTCAACGTG
AGCGTCGAGGACATTCTGTACGGCGATCACGAATGTGGGTCTCTGCTCCAGGACACCGCCCTGTATCTCG
TGGATGGCATGACCAACACAATCGAGAATGCCAGGCAGGGAGCCGCTAGAGTGACCTCTTGGCTGGGCAG
GCAGCTCAGCACAGCTGGAAAGCGCCTGGAGGGCCGGTCCAAAACCTGGTTTGGGGCCTACGCT<u>ATGGCC</u>
<u>CCAACCAGGCCACCCAGCCCAGTGACAAGACCTTGGCAGCACGTCGACGCCATTAAGGAGGCTCTGTCCC</u>

GM-CSF

<u>TGCTCAACAATTCTAACGATACCGCCGCTGTGATGAATGAAACAGTCGACGTGGTCTGCGAGATGTTCGA</u>
<u>TCCTCAGGAACCAACCTGCGTGCAGACAAGACTGAACCTCTACAAGCAGGGACTGAGGGGGAGCCTGACC</u>
<u>CGGCTCAAGTCCCCTCTGACACTGCTCGCCAAACACTATGAGCAGCATTGCCCACTGACCGAGGAAACAA</u>
<u>GCTGTGAAACCCAGTCCATCACATTCAAGTCTTTTAAAGACTCCCTGAATAAGTTCCTGTTTACCATCCC</u>

TTTTGATTGCTGGGGGCCTGTGAAGAAG*gactacaaagacgatgacgacaag****TAA***CTGATCATAATCAGC*
Flag **Stop**
CATACCACATTTGTAGAGGTTTTACTTGCTTTAAAAAACCTCCCACACCTCCCCCTGAACCTGAAACATA
SV40 terminator
AAATGAATGCAATTGTTGTTGTTAACTTGTTTATTGCAGCTTATAATGGTTACAAATAAAGCAATAGCAT
CACAAATTTCACAAATAAAGCATTTTTTTCACTGCATTCTAGTTGTGGTTTGTCCAAACTCATCAATGTA
*TCTTA***GGATCC**gcctctcgacgtatcgtcccgctcctcggaccccgtcaacgtgaccgtcgcctggttct
BamHI **PRV gG flanking downstream**
ttgacggcggccactgcaaggtgcccctcgtccaccgcgagtactacggctgccccggggacgccatgcc
ctccgtcgagacgtgcaccggcgggtactcgtacacccgcacgcgcatcgacaccctgatggagtacgcc
ctcgtgaacgccagcctcgtgctgcagcccgggttgtacgacgccggcctgtacatcgtcgtgctcgtct
ttggcgacgacgcctacctcggcaccgtctccctgtcggtggaggccaacctggactacccctgcggcat
gaagcacgggctcacgatcacccgccccggggccaccctcccacccatcgcccccacggccggcgaccac
cagcgctggcgcgggtgcttcccctcgaccgacgagggcgcctgggagaacgtgaccgccgccgagaagg
gcctgtccgacgactacgccgactactacgacgtgcacatcttccgctcggagtctgacgacgaggtcgt
ccacggcgatgcccccgaggcccccgagggcgaggaggtgaccgaggaggaggccgagctgacctccagc
gacctcgacaacatcgagatcgaggtcgtgggctctcccgccgctcccgtcgagggcgccggcgacggcg
aggagggcgcggggacgaggaggacgaggagctgacctccagcgacctcgacaacatcgagatcgaggt
cgtgggctcgcccgcggccgcccgcttcttcgccgcctccaccacccccgcgcccccacccgcgcggcc
gagatcacgaccatgaccacggtcaccaccgtgcggacgaccgaggaccccagcggcatcaccgactgcc
gccggagcgacttcgtctcgccctctgacatcttcgtgacccccaccggcagccccgccctgctcctggg
cttcctgggcagcgcgctcgcctcg**AAGCTT**– 3
HindIII’

Supplementary figure S3: Nucleotide sequence of PRV gGΔ CSFV E^{rns} GM-CSF insertion plasmid (pPRV gGΔ CSFV E^{rns}-GM-CSF-INS). The 4021 bp long nucleotide sequence for the PRV gGΔ CSFV E^{rns} GM-CSF Flag insertion was assembled from plasmid pPRV gGΔ and pCSFV E^{rns} GM-CSF chimeric gene cassette. The PRV gGΔ CSFV E^{rns} GM-CSF insertion plasmid consisted of the following (5’ to 3’): i) EcoRI restriction site (bold, underlined); ii) PRV gG flanking upstream (lower case); iii) Us3/Us4 polyadenylation signal (lower case, bold, italic); iv) KpnI restriction site (bold, underlined); v) CMV promotor sequence; vi) Kozak sequence (italic); vii) PRV gD signal (underlined); viii) CSFV E^{rns} coding sequence (highlighted); ix) GM-CSF coding sequence (underlined, highlighted); x) Flag epitope coding sequence (lower case, italic); xi) Stop codon (TAA) (bold, italic); xii) SV40 terminator (italic, underlined); xiii) BamHI restriction site (bold, underlined); xiv) PRV gG flanking downstream (lower case); HindIII restriction site (bold, underlined).

Supplementary table S1: Growth kinetics assay for PRV wild type and PRVtmv+. Virus titer (PFU/ml) (hours-post infection). PFU - Plaque forming units

Virus	Virus titer (PFU/ml) (hours-post infection)										
	0	1	3	6	8	12	18	24	30	36	44
PRV wild type	4.6275	3.2385	3.176	2.9375	4.6505	6.199	7.287	7.088	7.471	7.088	7.176
PRVtmv+	4.642	3.2385	2.699	3.272	5.088	5.8495	7.3495	7.565	7.398	7.1775	7.1505

Supplementary table S2: Rectal temperature

Body temperature in pigs (°C)
(Normal range – 38.67 to 39.78°C)
dpv - days-post vaccination

dpv	Control group (°C)					Fostera group(°C)					PRVtmv+ group (°C)				
	2311	2307	2301	2308	2314	2319	2316	2318	2305	2310	2320	2313	2304	2317	2301
0	39.44	39.05	38.56	39.17	39.22	39.33	39.78	39.67	39.39	39.67	38.05	38.44	39.67	38.33	39.44
2	39.44	39.78	39.33	39.56	39.61	39.33	39.67	39.39	39.05	39.83	39.44	39.78	39.11	37.78	39.22
4	39.94	40.05	39.05	39.56	39.44	39.78	39.5	39.61	39.44	39.44	39.16	39.11	39.61	39.16	39.78
6	39.94	40.28	39.44	38.94	39.44	39.78	39.33	40	40.22	40.05	39.66	39.67	38.61	38.94	39.5
8	38.33	38.78	38.89	38.61	39.11	38.89	39.44	39	38.89	39.66	39.05	39.5	39.16	39	39.83
15	38.72	39.5	38.67	39.5	37.78	39.11	38.78	39.67	39.17	39.78	39.44	39.89	39.83	39.05	39.94
21	38.17	39.56	38.44	38.72	39.11	39.67	39	39	39.56	38.83	38.72	39.17	39.16	38.83	39.5
32	39.17	39.5	38.83	39.33	38.89	39.44	39.61	39	39.28	39.11	39.05	39.05	39.33	39.05	38.56
39	38.78	39.39	39.33	39.39	39.28	39.05	39.78	39	39.39	38.89	38.78	39.05	38.944	38.94	39.56
45	38.17	38.5	39.17	39.56	39.28	39	39.28	39.11	39	38.67	39.56	39.5	39.11	38.83	38.39
49	39.39	39.33	39.28	39.39	39.5	39.5	39.56	39.28	38.83	38.39	39.05	39.61	39.44	39.11	39.56
53	37.78	39	37.39	37.78	37.61	39	38.22	39	38.28	37.78	38.55	39.11	38.61	38	39.22

Supplementary table S3: Body weight (in pounds)

Group	Animal	Body weight (in pounds) days-post vaccination				
		Day 0	Day 6	Day 32	Day 45	Day 53
Control	**2311**	38.8	49.2	63.8	75.2	77
	**2307**	39.5	41	53	58.6	62.2
	**2301**	32.6	34.8	45.2	50.6	54.2
	**2308**	27.2	39	50	59	62.8
	**2314**	44.2	45.8	57.4	59	63.8
Fostera	**2319**	36.8	34.6	46.2	54.8	58.6
	**2316**	38	36.6	52.4	58.2	61
	**2318**	42.8	43.4	60.4	71.4	80.8
	**2305**	35.8	34.6	45.6	51.6	48.2
	**2310**	39.4	41	51.2	53.2	56.1
PRVtmv+	**2320**	37	37.8	51	56.8	58.8
	**2313**	36	36.8	50.6	59.4	61.6
	**2304**	40.8	42.6	53	63.8	65.6
	**2317**	39	39.4	50.6	56.8	62.2
	**2302**	41.8	43.4	60.4	64.4	64.4

Supplementary table S4: PRVtmv+ vaccine virus replication in immunized pigs (nasal and tonsil swabs) - PRV-specific qPCR and virus isolation in swine kidney cells (in PRVtmv+ immunized pigs)

dpv – days post vaccination; NI – Not isolated

Animal	PRV qPCR – nasal swab				PRV isolation PFU/nasal swab			
	2 dpv	4 dpv	8 dpv	14 dpv	2 dpv	4 dpv	8 dpv	14 dpv
2320	12	7854	170	0	NI	NI	NI	NI
2313	1785.5	26826	0	0	NI	120	NI	NI
2304	22.5	19615	0	0	NI	30	NI	NI
2317	0	15774	794	0	NI	NI	NI	NI
2302	706.5	3994	0	0	NI	NI	NI	NI

Animal	PRV qPCR – tonsil swab				PRV isolation PFU/tonsil swab			
	2 dpv	4 dpv	8 dpv	14 dpv	2 dpv	4 dpv	8 dpv	14 dpv
2320	724	1649	179	0	60	15	NI	NI
2313	20222	148	77	0	810	NI	NI	NI
2304	908	354	322	0	45	NI	NI	NI
2317	3205	568	0	0	60	NI	NI	NI
2302	217	147	374	0	NI	NI	NI	NI

Supplementary table S5a: PRV-neutralizing antibody titer

Control group	PRV-neutralizing antibody titer – days-post vaccination (Days-post PCV2b challenge)						
	Day 0	Day 8	Day 15	Day 21	Day 32 (0 dpc)	Day 45 (13 dpc)	Day 54 (21 dpc)
**2311**	<4	<4	<4	<4	<4	<4	<4
**2307**	<4	<4	<4	<4	<4	<4	<4
**2301**	<4	<4	<4	<4	<4	<4	<4
**2308**	<4	<4	<4	<4	<4	<4	<4
**2314**	<4	<4	<4	<4	<4	<4	<4
**Average**	**<4**	**<4**	**<4**	**<4**	**<4**	**<4**	**<4**

PRVtmv+ group	PRV-neutralizing antibody titer – days-post vaccination (Days-post PCV2b challenge)						
	Day 0	Day 8	Day 15	Day 21	Day 32 (0 dpc)	Day 45 (13 dpc)	Day 54 (21 dpc)
**2320**	<4	6.68	76.34	12.62	26.5	25.84	13.26
**2313**	<4	23.35	47.07	14.33	57.21	79.87	29.59
**2304**	<4	<4	40	7.03	23.17	13.08	6.3
**#2317**	<4	5.74	24.84	<4	15	6.3	5.4
**#2302**	<4	5.65	10.02	<4	<4	<4	<4
**Average**	**<4**	**10.36**	**39.65**	**6.80**	**24.38**	**25.02**	**10.91**

Supplementary table S5b: PCV2b-neutralizing antibody titer

Group	Animal	PCV2b-neutralizing antibody titer – days-post vaccination (Days post PCV-2 challenge)						
		Day 0	Day 15	Day 21	Day 32 (Day 0)	Day 39 (Day 7)	Day 45 (Day 13)	Day 53 (Day 21)
Control	2311	<4	<4	<4	<4	<4	5.56	9.39
	2307	<4	<4	<4	<4	<4	<4	9.49
	2301	<4	<4	<4	<4	<4	<4	10.08
	2308	<4	<4	<4	<4	<4	<4	9.8
	2314	<4	<4	<4	<4	<4	5.55	25.36
Average		**<4**	**<4**	**<4**	**<4**	**<4**	**2.22**	**12.82**
Fostera	2319	<4	5.51	8.26	9.95	14.03	4.84	23.95
	2316	<4	10.87	5.52	16.25	12.19	5.27	42.68
	2318	<4	5.26	5.19	10.97	9.77	4.44	13.68
	2305	<4	10.65	7.79	20.21	14.73	7.08	15.28
	2310	<4	6.96	13.05	23.48	16.97	9.89	44.17
Average		**<4**	**7.85**	**7.97**	**16.17**	**13.54**	**6.30**	**27.95**
PRVtmv+	2320	<4	17.16	9.9	23.64	16.29	6.85	20.42
	2313	<4	18.81	10.28	25.95	16.80	5.97	39.24
	2304	<4	16.89	11.25	24.24	21.56	9.47	37.53
	2317	<4	6.96	5.49	14.82	4.94	4.97	41.79
	2302	<4	14	13.50	23.75	16.94	18.74	81.57
Average		**<4**	**14.76**	**10.01**	**22.48**	**15.31**	**9.2**	**44.11**

Supplementary table S5c: CSFV-neutralizing antibody titer

PRVtmv+ group	CSFV-specific neutralizing antibody titers (days-post vaccination)			
	0 dpv	15 dpv	21 dpv	32 dpv
**2320**	3	12	12	16
**2313**	<2	6	12	8
**2304**	<2	3	8	24
**2317**	<2	2	3	6
**2302**	<2	2	4	3
**Average**	**0**	**5**	**7.8**	**11.4**

Supplementary table S6: Percent changes in leukocytes and lymphocytes count. dpv – days-post vaccination; dpc – days-post challenge

Group	Animal	Leukocytes count ($x10^3/\mu l$)			Lymphocytes count ($x10^3/\mu l$)		
		32 dpv (0 dpc)	53 dpv (21 dpc)	% difference (0 and 21 dpc)	32 dpv (0 dpc)	53 dpv (21 dpc)	% difference (0 and 21 dpc)
Control	2311	18.3	13.7	-25.13	10.79	7.53	-30.21
	2307	20.8	11.5	-44.711	13.29	8.99	-32.35
	2301	20	15	-25	10.81	5.85	-45.88
	2308	18.2	15.9	-12.63	10.57	5.57	-47.30
	2314	22.8	13.7	-39.91	11.4	10.13	-11.14
Fostera	2319	20	15.2	-24	8.81	5.34	-39.38
	2316	20.8	17.7	-14.90	11.24	9.22	-17.97
	2318	12.6	16.7	32.53	10.42	11.17	7.19
	2305	21.6	13.7	-36.57	14.65	11.13	-24.02
	2310	17.9	17.2	-3.91	15.61	14.13	-9.48
PRVtmv+	2320	16.9	19.6	15.97	9.27	9.71	4.746
	2313	14.7	14.1	-4.08	11.21	13.49	20.33
	2304	11.1	16	44.14	8.86	8.17	-7.78
	2317	14.5	22	51.72	7.12	7.04	-1.12
	2302	17.7	22	24.29	9.21	11.73	27.36

Supplementary table S7: Fecal PCV2b shedding. PCV2b- capsid gene-specific qPCR assay

Group	Animal	Days post PCV-2 challenge (genome copies) (two replicates)							
		Day 0		Day 7		Day 13		Day 21	
Control	2311	-	-	-	-	11	14	16	9
	2307	-	-	-	-	-	-	-	-
	2301	-	-	-	-	-	-	-	-
	2308	-	-	-	-	37	34	-	-
	2314	-	-	-	-	-	-	-	-
Fostera	2319	-	-	-	-	-	-	-	-
	2316	-	-	-	-	-	-	-	-
	2318	-	-	-	-	-	-	-	-
	2305	-	-	-	-	-	-	-	-
	2310	-	-	-	-	-	-	-	-
PRVtmv+	2320	-	-	-	-	-	-	-	-
	2313	-	-	-	-	-	-	-	-
	2304	-	-	-	-	-	-	-	-
	2317	-	-	-	-	-	-	-	-
	2302	-	-	-	-				

Supplementary table S8a: PCV2b-viremia in serum. PCV2b- capsid gene-specific qPCR assay

Group	Animal	Days post PCV-2 challenge (genome copies) (two replicates)							
		Day 0		Day 7		Day 13		Day 21	
Control	2311	-	-	-	-	-	-	23	24
	2307	-	-	38	62	-	-	-	-
	2301	-	-	42	48	151	101	15	7
	2308	-	-	-	-	17	21	-	-
	2314	-	-	144	131	-	-	-	-
Fostera	2319	-	-	-	-	-	-	-	-
	2316	-	-	-	-	44	38	-	-
	2318	-	-	-	-	-	-	-	-
	2305	-	-	-	-	19	12	-	-
	2310	-	-	-	-	-	-	-	-
PRVtmv+	2320	-	-	-	-	-	-	-	-
	2313	-	-	-	-	-	-	-	-
	2304	-	-	-	-	-	-	-	-
	2317	-	-	-	-	-	-	-	-
	2302	-	-	-	-	-	-	-	-

Supplementary table S8b: PBMC-associated PCV2b viremia. PCV2b- capsid gene-specific qPCR assay

Group	Animal	Days post PCV-2 challenge (genome copies) PBMC-associated viremia (two replicates)							
		Day 0		Day 7		Day 13		Day 21	
Control	2311	-	-	-	-	-	-	5485	5863
	2307	-	-	223	312	-	-	596	407
	2301	-	-	872	787	2776	2855	14554	14013
	2308	-	-	-	-	1438	1605	912	674
	2314	-	-	783	801	602	455	843	1007
Fostera	2319	-	-	-	-	-	-	-	-
	2316	-	-	-	-	179	212	-	-
	2318	-	-	-	-	88	69	-	-
	2305	-	-	-	-	437	327	-	-
	2310	-	-	-	-	-	-	-	-
PRVtmv+	2320	-	-	-	-	-	-	-	-
	2313	-	-	-	-	-	-	-	-
	2304	-	-	-	-	-	-	-	-
	2317	-	-	-	-	-	-	-	-
	2302	-	-	-	-	-	-	-	-

Supplementary table S9: Quantification of PCV2b viral genome copies in pig's lymphoid tissues.
Normalized viral genome copies (per 10^6 cells); LN – lymph node

Group	Animal	PCV-2 – normalized mean viral genome copies (per 10^6 cells)					
		Tonsil	Mesenteric LN	Mediastinal LN	Cervical LN	Peyer's patches	Spleen
Control	2311	32	-	-	828	-	-
	2307	77	61	38	838	79	50
	2301	826	15	-	575	2503	-
	2308	106	-	-	140	-	-
	2314	589	742	71	1628	13189	5955

Group	Animal	PCV-2 normalized mean viral genome copies (per 10^6 cells)					
		Tonsil	Mesenteric LN	Mediastinal LN	Cervical LN	Peyer's patches	Spleen
Fostera	2319	-	-	-	-	-	-
	2316	-	-	-	-	-	-
	2318	-	-	-	-	-	-
	2305	23	-	37	21	-	-
	2310	-	-	58	7	861	-

Group	Animal	PCV-2 normalized mean viral genome copies (per 10^6 cells)					
		Tonsil	Mesenteric LN	Mediastinal LN	Cervical LN	Peyer's patches	Spleen
PRVtmv+	2320	-	-	-	-	-	-
	2313	-	-	-	-	-	-
	2304	-	-	-	-	-	-
	2317	-	-	-	-	-	-
	2302	-	-	22	-	-	-
